# Intimate Partner Violence during the COVID-19 Pandemic: A Review of the Phenomenon from Victims’ and Help Professionals’ Perspectives

**DOI:** 10.3390/ijerph18126204

**Published:** 2021-06-08

**Authors:** Giulia Lausi, Alessandra Pizzo, Clarissa Cricenti, Michela Baldi, Rita Desiderio, Anna Maria Giannini, Emanuela Mari

**Affiliations:** Department of Psychology, Sapienza University of Rome, 00185 Rome, Italy; alessandra.pizzo@uniroma1.it (A.P.); cricenticlarissa@gmail.com (C.C.); michela.baldi@uniroma1.it (M.B.); desiderio.ri@gmail.com (R.D.); annamaria.giannini@uniroma1.it (A.M.G.); e.mari@uniroma1.it (E.M.)

**Keywords:** stay at home, coronavirus, thematic synthesis, aggressive behavior, helping professions, psychological violence, lockdown, forced cohabitation, victimization

## Abstract

Social isolation is considered one of the main risk factors leading to intimate partner violence episodes; this evidence also emerged during the application of stay-at-home policies to contain the COVID-19 pandemic. For this reason, we aimed to collect data on intimate partner violence over the last year, comparing data reported by victims with data collected by help professionals. In accordance with PRISMA guidelines, through keywords related to abuse, pandemic and containment measures, 3174 articles were identified for screening. After full-text reading and risk of bias analysis, 19 studies were included, and a thematic synthesis was conducted according to two categories: “studies with victims” and “studies with help professionals”. The results of the present review showed that there were significant differences between the data provided by victims and the data collected by health care facilities and police departments; additionally, differences among different forms and severity of victimization emerged. The results have been discussed according to the literature; in particular, we reflected on how containment measures have apparently made it more difficult for victims to report, thus making the existence of the dark figure of crime even more salient.

## 1. Introduction

Domestic violence abuse (DVA) is a widespread public health problem [1,2] that includes different kinds of abuse, such as that upon elderly individuals and children in the family, while intimate partner violence (IPV) refers to violence by a current or former spouse or partner in an intimate relationship with the victim. IPV can be physical, psychological, sexual, or economic and can have negative health consequences on the victim [3,4,5,6].

Both geographical and social isolation may contribute to violence among people living together and the sequential consequences of a lack of social networks and support, a main protective factor in IPV episodes [7,8]. This aspect was severely challenged in 2020 and was characterized by a sudden and unexpected event: the identification of a new virus isolated in China and its subsequent spread, which led the World Health Organization (WHO) to declare a global pandemic in March of the same year [9]. Worldwide, several confinement measures have been taken to reduce the risk of infection; measures have differed among different countries, but all agreed on the advice (or, in some cases, orders) to stay at home (SAH), reduce mobility and increase social distancing across individuals [3,10]. The SAH policies were effective in limiting the spread of the virus but resulted in profound crises on several levels: public health, economic crisis, increased unemployment, and difficulties of forced cohabitation [11,12]; moreover, there has been an impact on society, which suffered from a lack of social support systems, and repercussions on, in particular, in intimate partner violence situations. This impact occurred both in new and in pre-existing IPV conditions [3,13,14,15,16,17]. According to some authors, forced cohabitation with an abusive partner may exacerbate individual and social vulnerability and limit coping skills and reliance on support networks [4,18].

DVA and IPV, during the different pandemic responses and situations of social isolation, were apparently associated with several factors: loss, bewilderment in facing an uncontrollable disaster, economic stress, increased exposure to risky and dysfunctional relationships, and lack of access to support systems, including health, law enforcement, and justice [2,18,19,20,21,22]. For instance, during previous pandemics, the fear of violence, as well as the fear of infection, seemed to lead women not to access health services, representing an important risk factor for recurring episodes of IPV during forced cohabitation [22]. Moreover, substance abuse, isolation and financial strain have been shown to be IPV risk factors that may be intensified during a pandemic period, as these factors can increase loneliness, increase psychological and financial stress, and increase the use of negative coping strategies, such as substance abuse [2,4,18]. Concerning the COVID-19 pandemic, early data collected on a global scale suggest that as isolation measures take effect, there has been a significant increase in IPV episodes since 2019 [4,13,23] regarding both reports and access to support services and websites [1,3,16,18,24,25]. Disruption of the individual’s social network during periods of quarantine and social distancing [16] may result in higher vulnerability for the victim and increased opportunities for the partner to commit violence [7,8]. Nonetheless, it is not possible to simply assume that the pandemic is the cause of the increase in violence, as this may be misleading and represent a justification for the perpetrator. For this reason, it is important to emphasize that an increase in violence is not related to the coronavirus per se as much as it is related to triggers such as isolation, anger, and stress that would seem to be intensified by the pandemic situation [17]. However, most data regarding the impact of COVID-19 social isolation measures on IPV have come from media and reports from victim support organizations [1,2,22,26].

As the restriction measures went into effect, the media highlighted a spike in IPV cases, sometimes with data that seemed to conflict. In Italy, for instance, during the SAH orders, a decrease in calls to the intimate partner violence hotline has been recorded; similar data have been found in Norway and in New York [27]. A possible explanation for this phenomenon may be the difficulty of victims seeking help, either because of social isolation that may amplify individual vulnerability and abusive behavior [1,4,10,28,29,30] or because of the coping strategies implemented by victims without incurring an increased risk [17].

Notwithstanding, several studies report different attitudes towards IPV reporting between victims and help professionals, both with regard to the perceived risk of reoffending [31] and the possibility of receiving/providing effective help [32,33]. Specifically, help professional reports—particularly those made by health providers [34,35,36]—seem to acknowledge a lower percentage of IPV cases than the victims, making it difficult to understand the real extent of this phenomenon. Nevertheless, health providers work through social services and shelters, allowing for systematic data collection and support and practical help for victims, both in recognizing the abusive situation and in getting out of it.

Crucially, the pandemic condition has drawn media attention to a phenomenon that should not be viewed through a causal filter but throughout its evolution. The fragmentary nature of data and news does not emphasize that IPV is a pattern of abusive behavior that stems from social and gender culture, nor is it a direct consequence of the COVID-19 emergency [13,17,28].

### Aim of the Study

Several reviews have examined the issue of IPV and its characteristics from the perspectives of victims, police, and healthcare facilities. However, to the best of our knowledge, no reviews have assessed the impact of the COVID-19 pandemic on IPV. Particularly, our study would like to fill the dark figure of crime issues through a multiperspective phenomenon analysis (i.e., victims, police, healthcare).

From these premises, the purpose of our review was (1) to collect research data on IPV during the COVID-19 pandemic to identify possible trends and (2) to highlight the features of this phenomenon by comparing data from victims (e.g., data collected from anonymous online surveys) and from help professionals. This includes all professionals (e.g., law enforcement officers, psychologists, doctors, health workers, educators) who activate supportive and helpful services in numerous fields, from social care and healthcare to security and prevention.

It is assumed that victims, because of the risk of infection, have preferred not to seek emergency care; moreover, hospitals and specialty facilities have limited access to support services for IPV victims, because of cases of staff contamination and because they are prioritizing the reception and care of those with COVID-19 [11,17].

## 2. Materials and Methods

This systematic review was performed according to the recommendations of the “Preferred Reporting Items for Systematic Reviews and Meta-Analyses” (PRISMA) [37,38,39,40]. The study was registered in the “International Prospective Register of Systematic Reviews” (PROSPERO) in March 2021 (CRD42020226376), and the detailed protocol is available upon request.

### 2.1. Inclusion Criteria

A systematic review of data concerning IPV (including physical, sexual, economic, or psychological abuse perpetrated by the partner) during the COVID-19 pandemic was conducted. To this aim, several electronic databases were screened: PubMed/MEDLINE, PsycINFO, Web of Science, Scopus, WHO (COVID-19 global literature on coronavirus disease), and CINAHL. In addition, a manual search of reference lists from relevant retrieved articles was performed.

The keywords used were related to abuse (“abuse” OR “violence” OR “IPV”), the ongoing COVID-19 pandemic (“Coronavirus” OR “COVID-19” OR “2019-ncov” OR “sars-cov-2” OR “pandemic”), and the containment measures adopted (“Lockdown” OR “lock-down” OR “isolation” OR “Quarantine” OR “social distanc*” OR “stay* home” OR “cohabitation”). After duplicate removal, 3174 records were selected for screening.

Articles selected included English, Italian, French, and Spanish languages and had to meet the following criteria: (1) article reported data on IPV; (2) violence reported was related to the ongoing COVID-19 pandemic; (3) articles were qualitative/quantitative/cross-sectional/cohort/non-report studies. Some studies were included even if they reported data on domestic violence, when it was specifically intended as any form of violence (i.e., physical, psychological, sexual, economic) perpetrated by a partner.

Articles were excluded if they addressed other forms of family violence, such as child or elder abuse, or if they reported witnessed or domestic violence in general. As a result, 3062 records were excluded based on title and abstract, and a total of 112 full-text articles were assessed for eligibility. Of these, 92 studies were excluded for the following reasons: incorrect focus, incorrect design or incorrect population. This resulted in a final number of 20 records reviewed.

The review process was as follows: three of the authors (AP, CC and RD) independently reviewed the titles and abstracts for relevance. In the next step, the authors read through the full articles and selected original studies that met the inclusion criteria. Disagreements were solved through discussion. The references in the selected articles were then checked to identify additional references of potential relevance. Pertinent editorials and reports that emerged from the literature search were retained as background papers. Subsequently, a methodological quality assessment and data abstraction were performed by three authors for all of the original studies selected.

The methodological criteria included adherence to the inclusion/exclusion criteria, appropriateness of the study population, and sampling and outcome definition and measurement.

### 2.2. Risk of Bias Assessment

**Figure 1:**
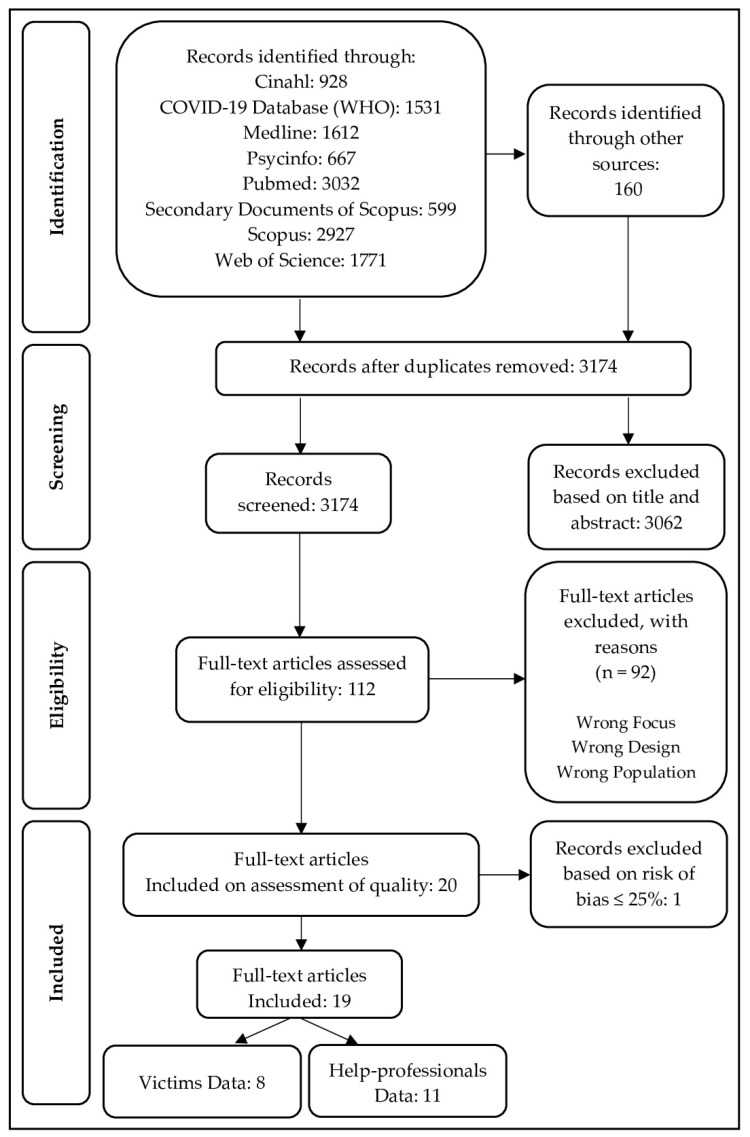
Flow chart of research articles selection, according to PRISMA criteria.

Two authors also performed a risk of bias assessment using two NIH quality assessment tools (https://www.nhlbi.nih.gov/, accessed on 20 January 2021): one for observational cohort and cross-sectional studies (*N* = 8) and one for before-after (pre-post) studies with no control group (*N* = 12). A third author performed a final review to ensure the appropriateness of the assessment procedure. A general quality rating assessment was performed to identify the general risk of bias amount: studies were rated as “good” if they showed ≥ 75% of positive answers to NIH tool questions (*N* = 11), they were rated as “fair” if they showed 50%–75% of positive answers to NIH tool questions (*N* = 5), and they were rated as “poor” if they showed 25%–50% of positive answers to NIH tool questions (*N* = 3). Studies reporting ≤ 25% of positive answers to the tool were rated as “very poor” and thus excluded (*N* = 1). Hence, 19 studies were reviewed (see Figure 1; Table 1 and Table 2).

### 2.3. Data Analysis

Because of the complexity of the phenomenon and its importance, a thematic synthesis was carried out to provide a first overview of the current state of the research on the topic [40]. Studies were categorized according to the target sample in “Studies with victims” (8) and “Studies with help professionals” (i.e., police officers, anti-violence center workers, health providers) (11). Data were treated as two independent studies to investigate whether there was a common trend in IPV reporting from the perspective of the victims as compared to that of the help professionals (Table 3 and Table 4).

## 3. Results

### 3.1. Studies with Victims

Most of the studies were cross-sectional and observational cohort studies. A total of 9752 participants joined IPV screening during the COVID-19 pandemic.

The studies were conducted on different continents: North America (3), Africa (2), Asia (2), and South America (1). Half of the studies (4) were conducted in the first months of the pandemic, during restrictive measures such as closing many activities and ordering people to stay at home; 12.5% of the studies (1) were conducted during the loosening of restrictive measures; 37.5% of the studies (3) involved the general population of the USA and, since each state implemented different measures for different periods, it was not possible to know the actual restrictive measure in place. However, as these were self-reported measures, all studies investigated the influence of pandemic-derived changes in IPV experiences.

#### 3.1.1. IPV Assessment

The following tools were used to assess IPV: ad hoc questionnaires (studies = 3) [41,42,43], validated instruments (studies = 4) [44,45,46,47] and interviews (study = 1) [48].

#### 3.1.2. Other Variables Assessment

Other variables that may have influenced the IPV assessment were also considered: COVID-19-related behaviors (studies = 3) [42,44,46] and COVID-19 cases (study = 1) [44]; depression and anxiety (studies = 2) [43,46]; general well-being and sociodemographic variables (studies = 4) [41,42,45,46] and addiction (studies = 3) [41,42,43].

#### 3.1.3. Results

Research findings showed that the effect of forced cohabitation led to increasing time spent together compared to before the COVID-19 period [47], which led to an increase in IPV on women [41,42,43,44,45,46,48]. A study conducted in Tunisia [43] showed an increase from 4.4% to 14.8% in the period before and during SAH policies. All examined data were the result of self-reported measures, showing an increase in IPV with a prevalence of verbal violence [41], together with emotional violence [46] and psychological violence [45], followed by economic [43], physical, and sexual violence [42,44,46,48]. A study conducted between the start of the lockdown and the loosening of restrictive measures showed the same results [46]. Notably, women in this study living with their husbands reported an increase in IPV in the periods before and during lockdown, with a higher prevalence of emotional violence (19.9%) than physical (6.5%) and sexual violence (3%).

These data seemed to increase when there was a COVID-19 positivity and therefore a forced quarantine with effects on the main dimensions of IPV: emotional, sexual and physical violence [44], as well as an increase in authors self-reporting physical violence [42]. This finding was strongly related to those who lost their jobs because of COVID-19 [44,47].

From the results that emerged in a study conducted at Johns Hopkins University in Baltimore (USA) [48], evidence suggests a correlation between the increase in life stressors (e.g., fear of losing one’s job, caregiving) and the increase in IPV incident frequency and severity because of the COVID-19 pandemic.

##### Gender Differences in IPV Victimization

Women who already experienced abuse before the pandemic event reported higher rates of violence (73%) than those who did not have a history of abuse (12%). Nearly 90% of the women who experienced violence during the SAH period did not seek help or report that abuse to authorities. None of the women who experienced emotional abuse reported it [43].

Violence during isolation has been associated with higher levels of depression, anxiety, stress, and Facebook addiction [43,48].

Two studies found a male prevalence of victimization during the pandemic period when compared to women [41,47].

##### Risk Factors for IPV Victimization

Among the most frequently found risk factors were age, educational level, and the possible presence of mental disorders [41,45]. According to a study from Nepal [41], younger respondents were more likely to experience violence; the same findings emerged in people with a lower educational level [41]. It was further found that participants with a previously diagnosed mental disorder were more likely to report having experienced physical and verbal violence [41]. Moreover, a study carried out in Ethiopia [45] showed how being a housekeeper and/or being married in an arranged union seemed to be associated with episodes of IPV from their respective husbands. In contrast, one study found that people who have never been married experienced IPV more than married people; furthermore, participants living alone with their spouse were more likely to experience violence, followed by those living with their friends [41].

A study by Nepalese Lalitpur University [41] found an increase in tobacco, drug, and alcohol use during SAH policies; this finding was also related to the number of victims of physical violence, thus substantiating the hypothesis of a causal relationship [41]. However, a study commissioned by the Inter-American Development Bank in Argentina [42] showed that even if an increase in drug and alcohol intake was found, a causal relationship in the increase in reported violence was not found. Finally, in only two studies [41,44], both IPV victimization and IPV perpetration were assessed. Both studies showed that those who had lost their jobs due to COVID-19 or tested positive for COVID-19 were more likely to perpetrate IPV [44]. Furthermore, during the lockdown, participants who said they perpetrated violence (18.2%) were more likely to have perpetrated both physical and psychological violence than those who only perpetrated one of the two forms [41].

### 3.2. Studies with Help-Professionals

Most of the studies evaluated data collected among help professionals (e.g., medical reports, calls to help lines, calls for police services) on IPV before and after the COVID-19 pandemic. Data were collected from North America (5), Central America (2), Europe (2), South America (1), and Oceania (1). A total of 81.8% of the studies (9) took into account data referring to the first months of the pandemic (coinciding with the introduction of the restrictive measures related to the closure of many activities and the order to stay at home), and 18.2% of the studies (2) also took into account data referring to the start of the loosening of restrictive measures.

#### 3.2.1. IPV Assessment

For data collection and comparison between the period of research interest (SAH restriction period vs. earlier years), the instruments referred to three contexts: the help lines (studies = 2) [49,50], the healthcare system (studies = 4) [28,29,51,52], and the police system (studies = 5) [10,15,20,53,54].

#### 3.2.2. Other Variables Assessment

Other variables that may have influenced the IPV assessment were also considered: health status and demographic variables (study = 1) [49], the severity of the injuries (study = 1) [28], and the change in traffic during the different time periods (study = 1) [53].

#### 3.2.3. Results

Research findings have shown that overall, the incident rate of calls to help services increased over time after the SAH policies [49,50,51,53], with several differences among countries. Data patterns also differed by whether the data were collected through access to healthcare facilities—through medical records or calls to help lines—or whether data were collected by police through reports and calls.

##### Data Collected from Healthcare Facilities

Data reporting access to healthcare services varied depending on the facilities that victims referred to; for instance, a study conducted in Peru [49] on calls to national help lines during the first months of the pandemic and the first period of the easing of restrictive measures, specifically from mid-March 2020 to July 2020, showed a 48% increase in calls to help lines for IPV compared to the same period in previous years. In particular, the call increase started in April (1.02 times) and accelerated in the following months (May: 1.58 times; June: 1.72 times; July: 2.12 times). Therefore, the loosening of restrictive measures did not appear to have affected the prevalence of IPV. The results also showed no differences among demographic groups, even considering the predetermined prevalence of IPV.

In contrast, data referring to hospital access and medical services differed. In fact, according to some studies [28,29], victims’ access to hospitals during the pandemic was lower on average than that during the same period in 2017–2019; however, the severity of injuries increased during the pandemic. In contrast, according to a study by the University of Chicago Medicine [51], the percentage of injuries from IPV increased after the SAH orders; it was observed that IPV incidents occurred more frequently during the nighttime period (6 pm–8 am) and during the week for those who spent most of their time at home. Moreover, comparing records between 2020 during SAH orders and previous years, a decrease in violence perpetrated by husbands toward wives was found, and there was also an increase in violence when perpetrated by nonmarital partners and other nonfamily members [29].

##### Data Collected from Police Records

Calls to the police increased from the previous year by 7.5%; furthermore, there was a more than two-fold increase in calls from people who had never sought help for IPV-related services [53]. Moreover, the data reported an increase in IPV calls and reports starting as early as the weeks before the official lockdown began, with an increase throughout the entire period of the containment measures for the spread of COVID-19 [52]. A study conducted in the UK [15] using data from police records and calls for police services collected during the early months of the pandemic and the first period of loosening restrictive measures showed an 8.1% increase in IPV during the pandemic period compared with the same period in previous years. In particular, in the first week of March, the number of calls and, to a lesser extent, the number of police reports, started to increase compared to previous years and remained higher throughout the lockdown period. Notably, the main increase in IPV calls was from third parties (e.g., neighbors) and high-density area reports; reports from third parties increased by 30% compared to previous years, while calls made by victims did not increase. Eventually, in the last weeks considered in the study (June), the general trend of increasing IPV began to decrease, as did calls made by third parties, although IPV was still significantly higher than in previous years. The role of third parties was also demonstrated in a study conducted in the USA [53], with an increased likelihood of reporting IPV via increased time spent at home because of the COVID-19 pandemic.

In contrast, several studies have found no significant differences in IPV during the SAH period; a research carried out on Los Angeles (USA) crime rates [20] found that virus containment policies caused no significant change in intimate partner aggression, regardless of the period examined (the first two weeks, March 4–16, or the entire period, March 4–28). Additionally, increased access to IPV calls was not observed, even though there was a change in the type of IPV call services requested from victims. In fact, there was an increase in IPV calls for psychological support services and a decrease in IPV calls for legal services [50]; in addition, a significant decrease in IPV (to −77%) was reported by Mexico City’s Attorney General’s Office [54]. Finally, a report released by the Australian NSW Bureau of Crime Statistics and Research [10] found that the number of IPV incidents did not significantly change between 2019 and 2020; nevertheless, victims could be unable to report because of home confinement with their perpetrator.

## 4. Discussion

This review allowed us to investigate the phenomenon of intimate partner violence from different perspectives. On the one hand, there were data from the victims themselves; on the other hand, there were data from reports and calls to healthcare facilities. This approach allowed a third observation, the comparison between the data provided by the authorities and the data from the victims, taking into account the difficulty of victims in reporting their abusive partners.

Data collected from this review showed an increase in the episodes of IPV reported by victims during SAH policies, particularly with a prevalence of verbal, emotional and psychological violence, followed by physical and sexual violence. It is worth noting that among the different forms of victimization, physical assault episodes decreased, although the severity of the assaults worsened among the victims [47]. Similar results have been found through data collected from healthcare facilities. In fact, victims’ access to hospitals was lower during the pandemic than in the same period in previous years; however, the severity of injuries increased during the pandemic [28,29]. This result might be explained by the perpetrators wanting to avoid hospitals, thus ensuring that the victimization was less harmful than that in normal conditions; moreover, the victims were not able to reach hospitals due to the spread of the virus and the at-home confinement with their abusers. Most IPV episodes occur through controlling behaviors of the abusive partner toward the victim. The implementation of SAH policies increased the difficulty of victims escaping the abusive behavior; it can also be assumed that SAH policies provided more control over the victims for the perpetrators, who had more knowledge of their movements [55,56,57,58,59]. This assumption was also supported by the data from this review; while victims reported more IPV episodes, the data collected by the police and healthcare services showed little change compared to previous periods [10,20], sometimes even significant declines [54], while a significant change seemed to emerge especially from those who had never sought help for IPV episodes [53].

Based on the data collected through the victims, it was found that physical violence was the one most associated with the increase in tobacco, drug and alcohol intake [41]; however, there is no certainty of a causal relationship between the two phenomena [42]. Additionally, it could be seen that most of the risk factors already found in the literature [60,61,62,63,64] were influential in the period of SAH policies, such as age, educational level, presence of mental disorders, or having previously experienced IPV [41,45]. In addition, having contracted the coronavirus or experienced a state of job uncertainty caused by the pandemic situation, with the subsequent increase in life stressors, seemed to represent new risk factors related to the specific time frame [44,47]. The association between coronavirus positivity and job loss because of COVID-19 and an increase in IPV emerged from both self-reports of victims and self-reports of IPV perpetrators [41,44].

With regard to perpetrator data, it should be noted that they were not sufficient to highlight an in-depth IPV perpetrator perspective; thus, we could not structure a specific discussion on this issue. In particular, there were no studies that specifically considered the perspective of offenders, especially regarding an increase or decrease in pre- and post-SAH violence, with a significant sample.

According to several studies [1,4,18,28], increasing the amount of time spent together with an abusive partner because of forced cohabitation has led to an exacerbation of a victim’s vulnerability and, moreover, to an abusive partner’s opportunity to perpetrate violence, failing to rely on social support, social networks, and the networking considered among the most important protective factors [7,8]. This result (that spending time together leads to increased vulnerability and therefore violence) is also in line with a UK study [15], which showed that despite a continuous increase in calls and police reports during the lockdown in June, coinciding with a loosening of restrictive measures, IPV started to decrease. However, the same result was not found in a study conducted in Peru [49]. It should be noted, however, that apart from the fact that these were calls to the help line and police calls/reports, the post lockdown period considered was very short and did not represent the focus of the study. In addition, the same study showed that the increase in IPV calls during the first months of the pandemic was mainly due to reports from neighbors rather than victims because COVID-19 caused people to stay at home more and they were more likely to notice worrying situations. This changed the victims’ seeking-help modality, making IPV calls for psychological support rather than legal support more accessible [50], while increasing the control of the abusive partner, which may have led to greater isolation for the victim. While there were mixed responses regarding the number of calls, both increases and decreases were attributed to an increase in IPV (e.g., fewer calls due to increased stalking and control by an abuser). Many participants consistently referred to the barriers survivors face when attempting to seek help, such as difficulties with new virtual platforms, closed court services that restrict access to necessary restraining orders, and closed shelters [48].

## 5. Conclusions

These results acquired considerable importance in addressing a phenomenon as complex as intimate partner violence. In fact, one of the main issues of data collection concerns the obscure number, i.e., the number of episodes of violence that are never reported, therefore affecting the estimations of the incidence of the phenomenon worldwide. On this subject, in a recent study conducted in Italy on the consequences of forced cohabitation during SAH orders, participants assumed an increase in episodes of IPV and an increase in separations as a result of forced cohabitation caused by restrictive measures in the territory. Although the data reported by research participants did not show a worsening within their daily lives [12], these findings provide a deeper understanding of the result shown by Freeman [10], who reported no change between the SAH period and the previous year but highlighted the increasing difficulty for victims to be able to report while living with their perpetrators.

The dark figure of crime is a pervasive limitation in domestic violence studies and, more specifically, with regard to intimate partner violence. In the interpretation of data from the reviewed studies, a substantial gap has already emerged between data reported by victims and those reported by professionals; although this finding supports the literature on the subject [65,66,67,68,69,70,71,72], the limitation that results in not being able to consider the data generalizable must be considered.

Beyond the limitation due to the obscure number, some inherent limitations in the present review must be considered. First, the wide range of methods and measures used for collecting and analyzing the data did not allow for more in-depth comparisons between the research examined; given the different research designs, this also led to the choice of using two different tools for the analysis of risk of bias.

Furthermore, it should be noted that most of the studies were carried out in the first few months following the onset of the pandemic, and there was no single restrictive measure for all countries, ranging from social distancing measures to more restrictive measures such as lockdowns. Despite this, some studies have considered data from the period of loosening of restrictive measures. However, the period following the first months of the pandemic has not been specifically taken into account. It would be interesting to evaluate these data in greater depth, for example with a future review of the literature using the restrictive measures implemented in the various countries as a variable, not only with respect to the official data but also with respect to what the victims self-reported. In fact, although some of the studies considered were conducted during the period of relaxation of the restrictive measures, the questions asked were focused on the violence experienced during the lockdown period, whereas it would be interesting to focus the study on the post lockdown period. Moreover, lacking sufficient data from all over the world, it was not possible to proceed with a comparison by area; the problem of gender-based violence is mainly due to cultural factors, and being able to highlight the different aspects from different parts of the world could allow more extensive and in-depth work. Future studies could analyze the data regarding IPV by comparing them with the coming years to identify whether the increase in episodes found in most of the studies examined will return to decrease or, on the contrary, the patterns will continue to change. Furthermore, intimate partner violence involves different dynamics than domestic violence and other forms of abuse; thus, future studies could investigate the phenomenon more extensively. Having a broader picture of violence could have practical implications for training health professionals to better support victims, mainly because of the constant change in these phenomena, both in how violence is perpetrated (e.g., increased control through electronic devices) and in how victims seek support and assistance.

Future research could further investigate the perspective of perpetrators to highlight the motivations and factors underlying the increase/decrease in violence during the pandemic period, as well as the types of violence most commonly used. As previously mentioned, distancing policies and orders to stay at home might have led to greater control over the victim by the partner, which might explain why in some situations a decrease in violence in the COVID-19 period and a decrease in severity were shown. Furthermore, it might be interesting to take gender differences into account in these terms. Highlighting the perspective of perpetrators could ultimately lead to a better understanding of the phenomenon and, consequently, to additional elements that could form the basis for combating the phenomenon of violence.

In terms of application, the results of this literature review could lead to the implementation of specific training for professionals (e.g., police, psychologists, and doctors), focusing on how to correctly receive requests for help, based on specific trainings with the use of role playing, both in person and on the help line. The training could also concern raising awareness and training with respect to the correct reading of signal or sentinel crimes, with the activation of standardized procedures at the national level. Raising awareness among the general population may also be worthwhile, as it has been found that the role of third parties, particularly neighbors, may be relevant in highlighting IPV episodes that otherwise remain unreported [15,53]. Awareness of IPV alarm signals and of increased risk in spending time with perpetrators in the general population may be an opportunity to decrease the dark figures of crime while increasing social support, as it is an important protective factor [7,8]. Therefore, developing interventions both on a large scale and in individual neighborhoods may contribute to preventing the IPV phenomenon.

It will be essential, however, to propose support and social reintegration projects for the victims, in light of the results of our study, whose objective will always be to put the needs of the victims at the center of the reintegration process. According to the available data, it would also seem useful to implement procedures that could make it easier to connect victims with institutions, especially in all cases where the victim has limited possibilities to communicate with the outside world. During the pandemic, progress was made regarding the use of “tele-health” [28,48,73,74]. Although there are still limitations to this procedure, it is a method that could help, even after the pandemic, all victims who are unable to visit a professional in person. It is also worth considering that the end of the pandemic will give victims a greater possibility to seek help and break out of the cycle of violence. This might mean making the availability of all those who help victims, from mental and physical health professionals to authorities, even more visible. Therefore, more effort might be needed to increase the possibilities for victims to meet these professionals.

## Figures and Tables

**Table 1:** Results of quality assessment of the cross-sectional studies (Victims Data).

Studies	1.	2.	3.	4.	5.	6. *	7. *	8. **	9.	10. ***	11.	12.	13. ****	14.	Quality Rating
[41]	Y	Y	Y	Y	NR	N	N	NA	N	NA	Y	Y	NA	Y	Good
[42]	Y	Y	CD	Y	NR	N	N	NA	N	NA	N	Y	NA	Y	Fair
[43]	Y	Y	Y	NR	NR	N	N	NA	N	NA	N	Y	NA	N	Poor
[44]	Y	N	Y	Y	NR	N	N	NA	Y	NA	Y	Y	NA	Y	Good
[45]	Y	Y	Y	Y	Y	N	N	NA	N	NA	Y	Y	NA	Y	Good
[46]	Y	Y	Y	Y	Y	N	N	NA	N	NA	Y	Y	NA	N	Good
[47]	Y	Y	Y	Y	NR	N	N	NA	N	NA	Y	Y	NA	CD	Fair
[48]	Y	N	CD	Y	NR	N	N	NA	N	NA	N	Y	NA	CD	Poor

Note. Quality of included studies was assessed using the National Institutes of Health (NIH) Quality Assessment tool for Observational Cohort and Cross-Sectional Studies (https://www.nhlbi.nih.gov/health-topics/study-quality-assessment-tools, accessed on 20 January 2021). 1. Was the research question or objective in this paper clearly stated? 2. Was the study population clearly specified and defined? 3. Was the participation rate of eligible persons at least 50%? 4. Were all the subjects selected or recruited from the same or similar populations (including the same time period)? Were inclusion and exclusion criteria for being in the study prespecified and applied uniformly to all participants? 5. Was a sample size justification, power description, or variance and effect estimates provided? 6. For the analyses in this paper, were the exposure(s) of interest measured prior to the outcome(s) being measured? 7. Was the timeframe sufficient so that one could reasonably expect to see an association between exposure and outcome if it existed? 8. For exposures that can vary in amount or level, did the study examine different levels of the exposure as related to the outcome (e.g., categories of exposure, or exposure measured as continuous variable)? 9. Were the exposure measures (independent variables) clearly defined, valid, reliable, and implemented consistently across all study participants? 10. Was the exposure(s) assessed more than once over time? 11. Were the outcome measures (dependent variables) clearly defined, valid, reliable, and implemented consistently across all study participants? 12. Were the outcome assessors blinded to the exposure status of participants? 13. Was loss to follow-up after baseline 20% or less? 14. Were key potential confounding variables measured and adjusted statistically for their impact on the relationship between exposure(s) and outcome(s)? CD: cannot determine; NA: not applicable; NR: not reported; N: no; Y: yes; *: For cross-sectional analyses, the answer to Questions 6 and 7 should be “no”; **: If there are only two possible exposures (yes/no), then this question should be given an “NA”, and it should not count negatively towards the quality rating; ***: Cross-sectional studies do not assess the exposure(s) more than one time, because of their own nature; ****: Cross-sectional studies do not require a follow-up.

**Table 2:** Results of quality assessment of the Before-After (Pre-Post) studies (Help-professionals Data).

Studies	1.	2.	3.	4. *	5. **	6.	7.	8.	9. ***	10.	11.	12. ****	Quality Rating
[10]	Y	Y	Y	CD	NA	Y	Y	Y	NA	N	N	NA	Good
[15]	Y	Y	Y	CD	NA	Y	Y	Y	NA	Y	Y	NA	Good
[20]	Y	Y	Y	CD	NA	Y	Y	Y	NA	Y	Y	NA	Good
[28]	Y	Y	N	CD	NA	N	Y	N	NA	Y	N	NA	Fair
[29]	Y	Y	N	CD	NA	Y	Y	NR	NA	Y	N	NA	Fair
[39]	Y	CD	Y	CD	NA	N	N	CD	NA	N	N	NA	Excluded
[49]	NR	N	Y	CD	NA	Y	Y	Y	NA	Y	Y	NA	Good
[50]	Y	Y	N	CD	NA	Y	Y	Y	NA	Y	Y	NA	Good
[51]	Y	Y	N	CD	NA	Y	Y	CD	NA	N	N	NA	Fair
[52]	NR	Y	N	CD	NA	Y	Y	NR	NA	N	N	NA	Poor
[53]	Y	Y	Y	CD	NA	Y	Y	Y	NA	Y	Y	NA	Good
[54]	Y	N	Y	CD	NA	Y	Y	Y	NA	Y	Y	NA	Good

Note. Quality of included studies was assessed using the National Institutes of Health (NIH) Quality Assessment tool for Before-After (Pre-Post) Studies with No Control Group (https://www.nhlbi.nih.gov/health-topics/study-quality-assessment-tools, accessed on 20 January 2021). 1. Was the study question or objective clearly stated? 2. Were eligibility/selection criteria for the study population prespecified and clearly described? 3. Were the participants in the study representative of those who would be eligible for the test/service/intervention in the general or clinical population of interest? 4. Were all eligible participants that met the prespecified entry criteria enrolled? 5. Was the sample size sufficiently large to provide confidence in the findings? 6. Was the test/service/intervention clearly described and delivered consistently across the study population? 7. Were the outcome measures prespecified, clearly defined, valid, reliable, and assessed consistently across all study participants? 8. Were the people assessing the outcomes blinded to the participants’ exposures/interventions? 9. Was the loss to follow-up after baseline 20% or less? Were those lost to follow-up accounted for in the analysis? 10. Did the statistical methods examine changes in outcome measures from before to after the intervention? Were statistical tests done that provided p values for the pre-to-post changes? 11. Were outcome measures of interest taken multiple times before the intervention and multiple times after the intervention (i.e., did they use an interrupted time-series design)? 12. If the intervention was conducted at a group level (e.g., a whole hospital, a community, etc.) did the statistical analysis take into account the use of individual-level data to determine effects at the group level?; CD: cannot determine; NA: not applicable; NR: not reported; N: no; Y: yes. *: Our studies reported data on IPV during lockdown, so we cannot determine if all of the participant enrolled were included according to prespecified entry criteria; **: I The sample taken into account for our study does not allow a measurement on confidence about his size; ***: Studies do not require a follow-up; ****: The studies we considered do not report any intervention; our finding is represented by the percentage of IPV reported itself.

**Table 3:** Coding Victim Data.

Authors	2020 SAH Period (*)	2020 Target Period	Country	Sample Details				Assessment of IPV	Assessment of Other Variables
				*N*	Mean Age (SD)	Gender	Ethnicity		
[41]	March–July	April–July	Nepal	556	25.93 (6.88)	F = 48.7% PnS = 0.4%	NA	AdHoc_Q	SA WHO-5
[42]	March– (May)	May	Argentina	1502	43.04 (11.43)	F	NA	AdHoc_Q	COVID-19_RB SC SEC
[43]	March– (June)	April–May	Tunisia	751	37 (8.2)	F	NA	AdHoc_Q	DASS-21 FBAS
[44]	-	March–May	USA	2045	46.63 (17.19)	F = 49.9% Ot = 1.5%	Af-Am = 11.9% As-Am = 2% Hs = 3.3% Ot = 20.1% Wt/Eu-Am = 62.6%	J-IPV	COVID19_CxS COVID-19_RB COVID-19 Status
[45]	(April–September)	April–May	Ethiopia	682	29.78 (5.78)	F	Ethiopian: Tigray = 99% Amara = 1%	WHO VAW	SDF
[46]	March–May	May–June	Bangladesh	2424	24.1 (4.8)	F	NA	WHO_MST	GAD-7 HFIAS
[47]	-	April	USA	1730	42 (13)	F = 59%	Hs = 8% Ot = 6% Wt = 73%	E-HITS	COVID-19_RB SDF
[48]	-	-	USA	45	-	F	Af As	Interview	-

Note. * Retrieved from https://www.imf.org/en/Topics/imf-and-covid19/Policy-Responses-to-COVID-19 (accessed on 26 May 2021), https://www.acaps.org/covid-19-government-measures-dataset (26 May 2021) and https://eu.usatoday.com/storytelling/coronavirus-reopening-america-map/ (26 May 2021); AdHoc_Q: Ad Hoc Questionnaire; Af: African; Am: American; As: Asian; COVID19_CxS: Covid19 cases per state; COVID19_RB: Covid19 Related Behaviours; DASS-21: Depression Anxiety and Stress Scales; E-HITS: Extended Hurt, Insulted, Threated and Scream; Eu: European; F: Female; FBAS: Facebook Bergen Addiction Scale; GAD-7: Generalised Anxiety Disorder; HFIAS: Household Food Insecurity Access Scale; HS: Hispanic; J-IPV: Jellinek Inventory for Assessing Partner Violence; Ot: Other; PnS: Prefer not Say; SA: Substance Abuse; SAH: Stay-at-Home; SC: Substance Consumption; SDF: Socio-Demographic Factor; SEC: Socio-Economic Characteristics; WHO-5: World Health Organization Wellbeing Index; WHO_MST: WHO Multicountry Survey Tool; WHO WAV: World Health Organization Violence Against Women; Wt: White.

**Table 4:** Coding Official Data.

Authors	2020 SAH Period (*)	2020 Target Period	Country (USA State)	Sample Details								Assessment of IPV	Assessment of Other Variable
				*N*		Mean Age (SD)		Gender		Ethnicity			
				Covid Period	No Covid Period	Covid Period	No Covid Period	Covid Period	No Covid Period	Covid Period	No Covid Period		
[10]	(March–May)	January–May	Australia	-	-	-	-	-	-	-	-	COPS	-
[15]	March–(May)	January–June	UK	PR: (2015-20): 385873 CP: (2019-20): 328385		-	-	-	-	-	-	PR/ CP	-
[20]	(March–May)	January–March	USA (CA)	47252	685615	-	-	-	-	-	-	PR	-
[28]	(April–June)	March–May	USA (MA)	62	342	37 (13)	41 (15)	F = 96%	F = 95%	AfAm = 8% Hs = 15% Ot = 12% Wt = 65%	AfAm = 36% Hs = 24% Ot = 14% Wt = 26%	EMR	ISS
[29]	March–April	March–April	USA (SC)	50	78	34.3 (12.4)	33.1 (15.6)	F = 38%	F = 47.4%	Bk = 22.0% Ot = 6% Ukn = 0% Wt = 72%	Bk = 28.2% Ot = 10.3% Ukn = 1.3% Wt = 60.3%	EDA	-
[49]	(March–May)	January–July	Peru	4075	-	-	-	-	-	-	-	CH	DSH
[50]	March–(May)	February–May	Mexico	-	-	-	-	F	-	-	-	CH	-
[51]	(March–May)	March–April	USA (IL)	62	88	32.4 (19.1)	36.9 (18.7)	F = 27.42%	F = 27.27%	AfAm = 80.7% As = 0% Cc = 12.9% Hs = 6.5%	AfAm = 75.0% As = 2.3% Cc = 13.6% Hs = 9.9%	EMR	-
[52]	(March–May)	March–April	UK	30	94	30.6	31	F = 10%	F = 4.26%	-	-	EMR	-
[53]	March–May	January–April	USA (IL)	-	-	-	-	-	-	-	-	PR/ CP/Arrests	L-Train SafeGraph Vehicular Traffic
[54]	March–(May)	January–May	Mexico	-	-	-	-	-	-	-	-	PR	-

Note. * Retrieved from https://www.imf.org/en/Topics/imf-and-covid19/Policy-Responses-to-COVID-19 (accessed on 26 May 2021), https://www.acaps.org/covid-19-government-measures-dataset (accessed on 26 May 2021) and https://eu.usatoday.com/storytelling/coronavirus-reopening-america-map/ (accessed on 26 May 2021); AfAm: African-American; As: Asian; Bk: Black; Cc: Caucasian; CH: Calls to the Helpline; COPS: Computerised Operational Policing System; CP: Calls for Police service; DSH: Demographic and Health Survey; EDA: Emergency Department Admissions; EMR: Electronic Medical Records; EuAm: European-American; F: Female; HS: Hispanic; ISS: Injury Severity Scale1; Ot: Other; PR: Police Records; SAH: Stay-at-Home; UKn: Unknown; Wt: White.

## Data Availability

The data presented in this study are available on request from the corresponding author.
